# Multiclass classification of microarray data samples with a reduced number of genes

**DOI:** 10.1186/1471-2105-12-59

**Published:** 2011-02-22

**Authors:** Elizabeth Tapia, Leonardo Ornella, Pilar Bulacio, Laura Angelone

**Affiliations:** 1 CIFASIS-Conicet Institute, Bv. 27 de Febrero 210 Bis, Rosario, Argentina; 2 Facultad de Cs. Exactas e Ingeniería, Riobamba 245 Bis, National University of Rosario, Argentina

## Abstract

**Background:**

Multiclass classification of microarray data samples with a reduced number of genes is a rich and challenging problem in Bioinformatics research. The problem gets harder as the number of classes is increased. In addition, the performance of most classifiers is tightly linked to the effectiveness of mandatory gene selection methods. Critical to gene selection is the availability of estimates about the maximum number of genes that can be handled by any classification algorithm. Lack of such estimates may lead to either computationally demanding explorations of a search space with thousands of dimensions or classification models based on gene sets of unrestricted size. In the former case, unbiased but possibly overfitted classification models may arise. In the latter case, biased classification models unable to support statistically significant findings may be obtained.

**Results:**

A novel bound on the maximum number of genes that can be handled by binary classifiers in binary mediated multiclass classification algorithms of microarray data samples is presented. The bound suggests that high-dimensional binary output domains might favor the existence of accurate and sparse binary mediated multiclass classifiers for microarray data samples.

**Conclusions:**

A comprehensive experimental work shows that the bound is indeed useful to induce accurate and sparse multiclass classifiers for microarray data samples.

## Background

A number of multiclass classification methods for microarray data have been developed in the recent years [1,2]. However, their ability to scale well to the number of classes and to provide accurate and sparse multiclass classification models essentially free of model selection-bias remain challenging issues [3,4]. Sparse multiclass classification models of microarray data samples are useful; they involve a reduced number of input genes and thus are easy to compute with and to interpret [5].

In this paper, a new gene selection method valid for binary mediated multiclass classification approaches of microarray data samples and able to implicitly model a gene selection sparsity constraint is presented. We rely on the use of output coding [6] methods allowing the binary reduction of *M*-multiclass classification into *n *binary classification tasks. We assume a model of independent genes, independent binary classifiers and a principle of information content equipartition among binary classifiers to derive a bound on the maximum number of genes that can be handled by binary classifiers in binary mediated multiclass classification approaches of microarray data samples. The derived bound scales with the inverse *n *thus providing a way to tackle the computational complexity of finding accurate and sparse multiclass classification models of microarray data samples: just increase the number *n *of binary classifiers and perform *bounded *optimum gene selection on lists of predictive genes for individual binary classifiers. In other words, the blessing face of dimensionality might be solution for the problem of accurate and sparse multiclass classifiers of microarray data samples; we just need to guarantee the induction of a large number *n *of independent binary classifiers. However, the induction of a large number *n *of independent binary classifiers by means of output coding methods may be hard to achieve when training data is scarce like in microarray data analysis. Hence, we may be forced to accept the best *n *with regard to the key independence factor [7,8] of general output coding methods. Just in case the best *n *is sufficiently large, the design of accurate and sparse multiclass classifiers of microarray data samples would be feasible.

Output coding embodies the design of well-known One Against All (OAA) [9] multiclass classifiers allowing the division of *M *- multiclass classification problems into *n *= *M *binary classification tasks, each binary task dealing with the problem of discriminating a given class against the others. A further generalization of OAA classifiers leads to the design of Error Correcting Output Coding (ECOC) classifiers [10,11] allowing the division of *M *- multiclass classification problems into *n *binary classification tasks, *n *being determined by the size of some error correcting code. ECOC classifiers can then be used to explore the feasibility of accurate and sparse multiclass classifiers of microarray data samples by letting *n *approach to infinity. In this paper, the recently introduced [12] class of ECOC classifiers based on LDPC codes [13] is considered. Hence, ECOC classifiers based on LDPC codes of size *n *up to ⌈15·*log*_2_*M*⌉ and OAA classifiers of size *n *= *M *are evaluated. For OAA as well as ECOC classifiers, binary linear Support Vector Machines (SVMs) [14] classifiers are assumed. For the purposes of selecting most important genes at core SVMs, univariate ranking information [15] based on the widely used S2N metric [16-18] is assumed. Using the above setting, a complete experimental protocol is presented for the design of accurate and sparse multiclass classifiers for microarray data samples essentially free of model selection-bias [19-22]. Our approach is evaluated on 8 benchmark microarray datasets. Experimental results confirm the feasibility of our proposed method.

## Results and Discussion

### An upper bound on the number of genes per binary classifier

How much information can a set of *p *independent genes convey about a set of *M *phenotypes? Being aware of such a fundamental limitation could be crucial in the design of accurate and sparse multiclass classifiers of microarray data samples. Let *S *be a microarray dataset comprising *q *samples from *M ≥ *3 classes, each sample defined by the gene expression measurements of *p *genes (*p *≫ *q*). Hence, the average information content per class sample in *S *can be upper bounded by *H_M _*= *log*_2_*M*.

In addition, let us assume that genes behave as a collection of *p *independent identically distributed *binary *random variables, i.e., a kind of probabilistic boolean model of gene expression is considered [23]. Hence, each gene is in state 1 (expressed) with probability *f *and in state 0 (not expressed) with probability 1 - *f*, each state representing gene activity above or below some threshold for an effect. Thus, in this model of gene expression, each gene conveys on average *H*(*f*) = - *f *· log_2 _*f *- (1 - *f*) · log_2_(1 - *f*) bits of information. Furthermore, let us assume an output coding strategy over *S *able to induce *n *independent binary datasets and correspondent binary classifiers. Hence, under a principle of information content equipartition, $H_{b}=H_{M}/n=\frac{log_{2}M}{n}$ bits of information will be available at each binary classifier. Finally, let us assume that each binary classifier is allowed to select a fraction *Q *of the complete set of genes. Hence, after the selection of *Q *· *p *genes, at most *Q *· *p *· *H*(*f*) bits of information will be available at each binary classifier and this quantity cannot exceed *H_b_*

(1) $$Q\cdot p\cdot H(f)\leq \frac{log_{2}M}{n}$$

Eq. 1 nicely estimates the maximum fraction of genes (*Q_max_*) that can be selected by any binary classifier in terms of main parameters characterizing any binary mediated multiclass classification problem plus an unknown parameter *f*. To estimate *f*, we now turn to the problem of estimating the probability *f *that a biased coin will come up with heads in a sequence of *q *independent coin tosses provided *k *heads have been observed. The maximum likelihood estimate of *f*, i.e., the value of *f *with the largest probability for the observed data, is given by *k*/*q*. To obtain *k*, we just need to count the number of expressed genes across the collection of *q *samples. However, aiming to obtain a more general bound, we would like to avoid overwhelming data dependent counts. If we further assume that averages of gene expression over a sufficiently large population of individuals are equal to averages over many genes, i.e., an ergodic behavior of genes [24] is considered, the fractional *f *should equal the fraction of genes k*//*p *that are expressed at any individual. Assuming that k*/*p *< 0.5 (otherwise not expressed genes can be considered) and recalling that *H *(*f*) is a monotonic increasing function in [0, 0.5], we get *H*(*k/q*) ≈ *H*(k*/*p*) ≥ *H*(1/*p*) and the following *Q *upper bound (*Q_max_*) can be derived

(2) $$Q_{max}\approx \;\frac{\log_{2}M}{p\cdot n\cdot H(1/p)}$$

**Figure 1:**
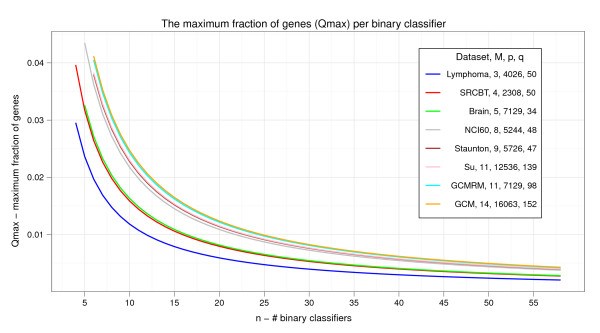
**The maximum fraction of genes per binary classifier**. The maximum fraction of genes *Q_max _*that can be handled by core binary classifiers in binary mediated multiclass classification of microarray data samples involving *p *genes and *q *samples, *p *> >*q*. Multiclass classifiers for *M *≥ 3 classes built from *n *binary classifiers, *n *≥ ⌈*log*_2_*M*⌉ + 2, are considered. *Q_max _*is evaluated on the 8 benchmark microarray datasets used in this paper (Lymphoma, SRCBT, Brain, NCI60, Staunton, Su, GCMRM and GCM).

Overall, Eq. 2 suggests that the computational complexity of finding sparse multiclass classifiers of microarray data samples could be overcome with the induction of a large number *n *of *independent *binary classifiers, a requirement which gets easier to satisfy as the number of training samples increases. The evolution of *Q_max _*with respect to *n *on benchmark microarray datasets used in this paper is shown in Figure 1. Before moving onto the next subsection, we notice that a more formal derivation of *Q_max _*is given in the Appendix.

### Bounded optimum S2N gene selection

For a fixed *n*, we now face the problem of finding the optimum number of genes in the list of top p* *Q_max_*(*n*) most discriminative genes for each binary classifier. Such optimum will follow from a partial search scheme and thus, we provide no guarantee of identifying the optimal gene set [25]. But as *n *increases, finding such optimum implies finding a sparse representation of a high dimensional feature space from a small number of training samples. Because sparsity is key structural property of most genomic studies involving disease classification, we conjecture that the proposed gene selection method could indeed be a solution for the problem of designing accurate and sparse multiclass classifiers of microarray data samples.

Letting *n *approach to infinity cannot be realized in practice. Hence, some bounded exploration of the *n *dimension must be assumed in advance. In this paper, the exploration of *n *dimension from *n_min _*= ⌈log_2_*M*⌉ + 2 up to *n_max _*= ⌈15·*log*_2_*M*⌉ is considered. Notice that *n *= ⌈15·*log*_2_*M*⌉ + 1 is not considered; it would entail the use of parity codes only able to detect (but not correct) binary classifiers errors. For practical *n *ranges, the exhaustive exploration of p* *Q_max_*(*n*) most important genes for each binary classifier may still be too computationally demanding. Thus, a multi-scale resolution approach for the *Q*-dimension was devised. Firstly, the *Q *dimension was coarsely quantized with a base 10 logarithmic scale, i.e., *Q *∈ [0.001, 0.01, 0.1, 1] was assumed. Secondly, each logarithmic segment, except the last one, was linearly quantized into 10 equal parts; the last logarithmic segment was quantized into 100 equal parts. Finally, genes at each binary classifier were ranked according to their *S*2*N *value (see Methods for details) with respect to the response variable and mapped to the formerly quantized *Q*-dimension for further selection. As a result, for a fixed computational budget, more computational effort can be put into the exploration of highly discriminative genes, i.e., top ranking genes, than into those of poor discriminative power.

### Results on Real Data

We first note that the application of the Shapiro-Wilk test to the empirical distributions of performance measures (classification error, overall fraction of selected genes and gene selection stability) of either ECOC or OAA classifiers frequently rejected the null hypothesis of normally distributed data at the 0.05 *α *level of significance, thus justifying the use of the more conservative Kolmogorov-Smirnov (KS) and Mann-Whitney (MW) U tests.

Table 1 shows the classification performance of OAA and ECOC classifiers of size *n *up to ⌈*η*·*log*_2_*M*⌉ (*η *= 5, 10, 15) over 200 Montecarlo 4:1 train-test partitions. Despite the *η *choice, ECOC and OAA classifiers attain comparable classification performance in 5 out of 8 datasets (*p *> 0.3, two-sided KS tests). Stochastic orderings favorable to OAA classifiers are observed in the SRCBT, NCI60 and GCM datasets (*p *< 0.05, one-sided KS tests; one-side MW tests consistent). In particular, OAA classifiers perform remarkably well on the hard NCI60 and GCM datasets.

**Table 1:** The classification performance of OAA and ECOC classifiers

						p-values^a^	
					
Dataset	M	n	Error-ECOC(F)	Error-OAA(G)	*F *≠ *G*	*F *<*G*	MW
200 Montecarlo 4:1 train-test partitions at *η *= 5							

Lymphoma	3	NA	NA	0	NA	NA	-
SRCBT	4	9	0	0	0.00437	0.00219	0.99682
Brain	5	9	0.1250	0.1250	0.98741	-	-
NCI60	8	9	0.3077	0.2308	0.02222	0.01111	0.99682
Staunton	9	12	0.4615	0.4615	0.71123	-	-
GCM RM	11	11	0	0	0.39273	-	-
Su	11	13	0.0857	0.0857	0.92282	-	-
GCM	14	12	0.3625	0.2863	9.99e-16	4.76e-16	1

200 Montecarlo 4:1 train-test partitions at *η *= 10							

Lymphoma	3	11	0	0	0.98741	-	-
SRCBT	4	9	0	0	0.00307	0.00153	0.99999
Brain	5	15	0.1250	0.1250	0.99970	-	-
NCI60	8	14	0.3077	0.2308	0.00213	0.00106	0.99996
Staunton	9	19	0.4615	0.4615	0.79201	-	-
GCM RM	11	12	0	0	0.79201	-	-
Su	11	17	0.0857	0.0857	0.32750	-	-
GCM	14	12	0.3624	0.2863	9.99e-16	4.76e-16	1

200 Montecarlo 4:1 train-test partitions at *η *= 15							

Lymphoma	3	11	0	0	0.98741	-	-
SRCBT	4	9	0	0	0.00307	0.00153	0.99999
Brain	5	18	0.125	0.125	0.99999	-	-
NCI60	8	16	0.3077	0.2308	0.00045	0.00022	0.99999
Staunton	9	19	0.4615	0.4615	0.62717	-	-
GCM RM	11	12	0	0	0.96394	-	-
Su	11	17	0.0857	0.0857	0.46532	-	-
GCM	14	12	0.3666	0.2863	< 2.2e-16	< 2.2e-16	1

The classification performance of ECOC classifiers of size at most ⌈*η*·*log*_2_*M*⌉ and OAA classifiers under bounded optimum *S*2*N *gene selection over 200 runs of Montecarlo 4:1 train-test partitions. *M *and *n *respectively denote the median number of binary classifiers at ECOC and OAA classifiers. Error-ECOC and Error-OAA respectively denote the median classification errors attained by ECOC and OAA classifiers. Error-ECOC and Error-OAA are denoted as F and G for purposes of KS tests, respectively.

^a ^p-values of two-sided KS tests, one-sided KS tests and one-sided MW tests. The alternative hypothesis of two-sided KS tests is "the error of ECOC classifiers is different from that of OAA classifiers", i.e., the relationship between CDFs is *F *≠ *G*. The alternative hypothesis for one sided KS tests is "the error of ECOC classifiers is greater than that OAA classifiers", i.e., the relationship between CDFs is *F *<*G*. The alternative hypothesis of one sided MW tests is "the error of ECOC classifiers is less than that of OAA classifiers".

Table 2 shows the overall number of genes selected by OAA and ECOC classifiers of size *n *up to ⌈*η*·*log*_2_*M*⌉ (*η *= 5, 10, 15) under bounded optimum *S*2*N *gene selection over 200 Montecarlo 4:1 train-test partitions. Moving from *η *= 5 to *η *= 15 gradually reduces the dimensionality of ECOC classifiers. The strongest reduction effect occurs when moving from *η *= 5 to *η *= 10, suggesting *η *= 10 as a practical upper limit for the exploration of the *n *dimension with ECOC classifiers. However, the extent of ECOC dimensionality reductions are insufficient to improve naive OAA classifiers. Despite the *η *choice, significant differences in the number of genes selected by ECOC and OAA classifiers are observed in all datasets (*p *< 0.05, two-sided KS tests). Stochastic orderings favorable to ECOC classifiers are observed in the Lymphoma and NCI60 datasets (*p *> 0.2, one-sided KS tests; *p *< 0.01, one-sided MW tests).

**Table 2:** The overall number of genes selected by OAA and ECOC classifiers

								p-values^a^	
							
Dataset	M	N	B-ECOC	B-OAA	G-ECOC(F)	G-OAA(G)	*F *≠ *G*	*F *<*G*	MW
200 Montecarlo 4:1 train-test partitions at *η *= 5									

Lymphoma	3	NA	NA	4	NA	22	NA	NA	NA
SRCBT	4	9	14.22	6	37	23	< 2.2e-16	< 2.2e-16	1
Brain	5	9	28.1	19	177	109.5	5.08e-05	2.54e-05	0.99975
NCI60	8	9	45.11	34	310	326	9.31e-07	0.27804	0.07651
Staunton	9	12	46	34.11	387	296	9.91e-08	4.95e-08	0.99993
GCM RM	11	11	142	36	800	365.5	< 2.2e-16	2.76e-08	1
Su	11	13	126	62	1056	916	5.36e-12	1.15e-24	0.99978
GCM	14	12	322	128	2096	1406	< 2.2e-16	< 2.2e-16	1

200 Montecarlo 4:1 train-test partitions at *η *= 10									

Lymphoma	3	11	4.27	4	12	22	5.52e-08	1	9.85e-09
SRCBT	4	9	12.22	6	33	23	< 2.2e-16	< 2.2e-16	1
Brain	5	15	16.16	19	109.5	109.5	0.03970	0.01984	0.54495
NCI60	8	14	42.12	39	286.5	326	9.31e-07	0.95599	0.00105
Staunton	9	19	40.03	34.11	381.5	296	6.95e-10	3.48e-10	0.99997
GCM RM	11	12	72	36	570	365.5	< 2.2e-16	1.66e-19	1
Su	11	17	112	62	940	916	1.82e-10	9.11e-11	0.98387
GCM	14	12	322	128	2078	1406	< 2.2e-16	< 2.2e-16	1

200 Montecarlo 4:1 train-test partitions at *η *= 15									

Lymphoma	3	11	4.26	4	12	22	3.05e-08	1	3.85e-09
SRCBT	4	9	12.22	6	33	23	< 2.2e-16	< 2.2e-16	1
Brain	5	18	16.06	19	105	109.5	0.03970	0.01984	0.15586
NCI60	8	16	36.15	39	251	326	9.31e-07	1	3.23e-05
Staunton	9	19	34.09	34.11	373.5	296	4.81e-09	2.41e-09	0.99989
GCM RM	11	12	72	36	561	365.5	< 2.2e-16	1.66e-19	1
Su	11	17	112	62	924.5	916	1.34e-09	6.69e-10	0.97006
GCM	14	12	322	128	2066	1406	< 2.2e-16	< 2.2e-16	1

The number of genes selected by OAA and ECOC classifiers of size at most ⌈*η*·*log*_2_*M*⌉ under bounded optimum *S*2*N *gene selection over 200 Montecarlo 4:1 train-test partitions. *M *and *n *respectively denote the median number of binary classifiers at OAA and ECOC classifiers. B-ECOC and B-OAA respectively denote the median number of genes per binary SVM at ECOC and OAA classifiers. G-ECOC and G-OAA respectively denote the median *overall *number of genes selected at ECOC and OAA classifiers. G-ECOC and G-OAA are denoted as F and G for purposes of KS tests, respectively.

^a ^p-values of two-sided KS tests, one-sided KS tests and one-sided MW tests. The alternative hypothesis of two-sided KS tests is "the number of genes selected by ECOC classifiers (F) is different from that of OAA classifiers (G)", i.e., the relationship between corresponding CDFs is *F *≠ *G*. The alternative hypothesis for one sided KS tests is "the number of genes selected by ECOC classifiers (F) is greater than that OAA classifiers (G)", i.e., the relationship between corresponding CDFs is *F *<*G*. The alternative hypothesis of one-sided MW tests is "the median number of genes selected by ECOC classifiers is less than that of OAA classifiers".

Table 3 shows the stability of gene selection attained by OAA and ECOC classifiers of size up to ⌈*η*·*log*_2_*M*⌉ (*η *= 5, 10, 15) under bounded optimum *S*2*N *gene selection over 200 Montecarlo 4:1 train-test partitions. Despite the *η *choice, significant differences in the stability of gene selection attained by ECOC and OAA classifiers are observed (*p *< 2.2*e *- 16, two-sided KS tests). Stochastic orderings favorable to ECOC classifiers are observed in Lymphoma, SRCBT and Su datasets (*p *> 0.9, one-sided KS tests; *p *< 2.2*e - *16, one sided MW tests); ambiguous orderings are observed in the Brain, GCM RM and GCM datasets. Remarkably, the stability of gene selection attained by ECOC classifiers is only slightly reduced when moving from *η *= 5 to *η *= 15.

**Table 3:** The stability of gene selection attained by OAA and ECOC classifiers

						p-values^a^	
					
Dataset	M	n	S-ECOC(F)	S-OAA(G)	*F *≠ *G*	*F *>*G*	MW
200 Montecarlo 4:1 train-test partitions at *η *= 5							

Lymphoma	3	NA	NA	0.5539	NA	NA	NA
SRCBT	4	9	0.6835	0.5652	< 2.2e-16	0.99979	< 2.2e-16
Brain	5	9	0.4643	0.4315	< 2.2e-16	0.02363	< 2.2e-16
NCI60	8	9	0.4313	0.4365	< 2.2e-16	< 2.2e-16	1
Staunton	9	12	0.4129	0.4119	< 2.2e-16	< 2.2e-16	0.73628
GCM RM	11	11	0.6043	0.6143	< 2.2e-16	< 2.2e-16^b^	< 2.2e-16
Su	11	13	0.6286	0.5461	< 2.2e-16	0.99594	< 2.2e-16
GCM	14	12	0.6783	0.5886	< 2.2e-16	1	< 2.2e-16

200 Montecarlo 4:1 train-test partitions at *η *= 10							

Lymphoma	3	11	0.6093	0.5539	< 2.2e-16	1	< 2.2e-16
SRCBT	4	9	0.6745	0.5652	< 2.2e-16	1	< 2.2e-16
Brain	5	15	0.4582	0.4315	< 2.2e-16	0.00213^b^	< 2.2e-16
NCI60	8	14	0.4234	0.4365	< 2.2e-16	< 2.2e-16	1
Staunton	9	19	0.4185	0.4119	< 2.2e-16	< 2.2e-16	5.93e-07
GCM RM	11	12	0.6112	0.6143	< 2.2e-16	6.83e-08^b^	< 2.2e-16
Su	11	17	0.6423	0.5461	< 2.2e-16	0.99154	< 2.2e-16
GCM	14	12	0.6650	0.5886	< 2.2e-16	0.42216	< 2.2e-16

200 Montecarlo 4:1 train-test partitions at *η *= 15							

Lymphoma	3	11	0.6093	0.5539	< 2.2e-16	1	< 2.2e-16
SRCBT	4	9	0.6740	0.5652	< 2.2e-16	1	< 2.2e-16
Brain	5	18	0.4591	0.4315	< 2.2e-16	0.00165^b^	< 2.2e-16
NCI60	8	16	0.4170	0.4365	< 2.2e-16	< 2.2e-16	1
Staunton	9	19	0.4168	0.4119	< 2.2e-16	< 2.2e-16	0.02409
GCM RM	11	12	0.6124	0.6143	< 2.2e-16	8.46e-05^b^	< 2.2e-16
Su	11	17	0.6405	0.5461	< 2.2e-16	0.99154	< 2.2e-16
GCM	14	12	0.6578	0.5886	< 2.2e-16	0.03809^b^	< 2.2e-16

The stability of gene selection attained by ECOC classifiers of size at most ⌈*η*·*log*_2_*M*⌉ and OAA classifiers under bounded optimum *S*2*N *gene selection over 200 Montecarlo 4:1 train-test partitions. *M *and *n *respectively denote the median number of binary classifiers at OAA and ECOC classifiers. S-ECOC and S-OAA respectively denote the stability of gene selection attained by ECOC and OAA classifiers measured by the Salton's coefficient. S-ECOC and S-OAA are denoted as F and G for purposes of KS tests, respectively.

^a ^p-values of two-sided KS tests, one-sided KS tests and one-sided MW tests. The alternative hypothesis of two-sided KS tests is "the stability of gene selection in ECOC classifiers (F) is different from that in OAA classifiers (G)", i.e., the relationship between corresponding CDFs is *F *≠ *G*. The alternative hypothesis for one sided KS tests is "the stability of gene selection in ECOC classifiers (F) is lower than that OAA classifiers (G)", i.e., the relationship between corresponding CDFs is *F *>*G*. The alternative hypothesis of one-sided MW tests is "the median stability of gene selection in ECOC classifiers is higher than that of OAA classifiers".

^b ^Difficult to definitely compare. Highly significant p-values for both one-sided KS tests.

For the sake of completeness, we also report the performance of OAA and ECOC classifiers of size at most ⌈*η*·*log*_2_*M*⌉ (*η *= 5, 10, 15) on two benchmark microarray datasets with a public train-test partition (see Table 4). Results agree with observed trends of the classification error in Montecarlo evaluations. Although both ECOC and OAA classifiers seem to be highly effective in the GCMRM dataset, suggesting that ECOC classifiers may be worthy of exploring in such case, only OAA classifiers perform well on the GCM dataset. Since the GCMRM dataset is just a subsample of the GCM dataset to which a more robust preprocessing protocol has been applied, so that fewer samples, fewer classes and fewer genes than in the original dataset are involved, these results raise the question to what extent specific preprocessing protocols could be a affecting the strength of gene selection attainable with ECOC classifiers.

**Table 4:** The performance of OAA and ECOC classifiers on train-test partitions

Dataset	M	n	G-ECOC	G-OAA	Error-ECOC	Error-OAA
			***η *= 5, 10, 15**			

GCM RM	11	10	926	1260	0.1852	0.1852
GCM	14	20	1314	423	0.4782	0.3043

The performance of OAA and ECOC classifiers of size at most ⌈*η*·*log*_2_*M*⌉ on benchmark microarray datasets under bounded optimum *S*2*N *gene selection and a public train-test partition. *M *and *n *respectively denote the number of binary classifiers used by OAA and ECOC classifiers. G-OAA and G-ECOC respectively denote the *overall *number of genes selected by OAA and ECOC classifiers. Error-OAA and Error-ECOC respectively denote the classification error attained by OAA and ECOC classifiers.

## Conclusions

The divide and conquer approach to the design of multiclass classifiers for microarray data samples which we have presented offers the hope that accurate and sparse multiclass classifiers can be constructed without incurring in undesirable forms of gene selection bias hidden in the selection of optimal gene subsets of restricted or unrestricted size [26]. Generalized binary reductions of *M*-multiclass classification problems into *n *binary classification tasks and bounded explorations of resulting gene spaces are advised to accomplish this objective. At each binary classifier, the maximum number of genes that can be selected scales with the inverse of *n*, thus providing a way to accomplish optimum gene selection at affordable computational costs, provided *n *is sufficiently large.

In this paper, the power of OAA and ECOC binary reductions in the design of accurate and sparse multiclass classifiers for microarray data samples has been evaluated. Without loss of generality, we have restricted ourselves to the class of ECOC classifiers based on LDPC codes, linear SVM binary classifiers and univariate *S*2*N *gene selection. Experimental results show that dimensionality exchange between input and output domains of binary mediated multiclass classifiers of microarray data samples is indeed possible: the larger the size of candidate ECOC classifiers, the greater the chance of selecting smaller sets of genes. Although promising, the dimensionality reduction performance exhibited by ECOC (LDPC) classifiers is not enough to definitely improve naive OAA classifiers, which remain the best practical option.

From an overall view, experimental results suggest that improving the dimensionality reduction ratio of OAA classifiers with ECOC classifiers may not be as easy as it seems. We note, however, that a consensus approach to gene selection and classification on a set of diverse ECOC classifiers under bounded optimum gene selection could finally boost their dimensionality reduction factor beyond that of OAA classifiers. Briefiy, provided individual ECOC solutions are good enough compared to OAA classifiers, a consensus approach to gene selection on a set of diverse ECOC classifiers should preserve most relevant genes and reject a great proportion of irrelevant ones. Since ECOC classifiers based on LDPC codes seem to be closely related neighbors of OAA counterparts, this hypothesis will be focus of future research. Finally, further dimensionality reduction improvements may still be attainable with more elaborated forms of gene selection like SVM-RFE [27].

Overall, our results provide evidence that bounded optimum gene selection in high dimensional binary output domains induced by either OAA or ECOC classifiers may be a solution for the problem of accurate multiclass classification of microarray data samples based on a reduced number of genes.

## Methods

To keep the paper self-contained in this section, we would like to briefiy review the design of ECOC classifiers based on LDPC codes. Then we proceed to describe benchmark microarray data and main points of our experimental protocol. The introduction of error correcting codes in the design of ECOC classifiers aims the automatic recovery of binary classifiers errors leading to erroneous multiclass predictions. For this purpose, an ECOC code must be first defined. An ECOC code is a binary matrix of size *M *by *n*, the *i*-*th *row defining the binary encoding for the *i*-*th *class label, *i *= 1,..., *M*, and the *j*-*th *column defining the binary split to be learn by the *j*-*th *core binary classifier, *j *= 1,..., *n*. Since codewords of length $n=\frac{\left\lceil \left(log_{2}M\right)\right\rceil }{R}$, 0 <*R *< 1, are required for redundantly encoding *k *= ⌈*log*_2_*M*⌉ bits of useful class label information, ECOC classifiers entail output designs of logarithmic complexity with respect to *M*, which can be an advantage when *M *is rather large [28]. As noted by [29], ECOC classifiers based on random ECOC codes are asymptotically Bayes Optimal, i.e., they approximate the minimum possible misclassification error, provided core binary classifiers are Bayes classifiers themselves. As noted by [30], the SVM paradigm efficiently approximates the Bayes classification rule. Hence, core binary classifiers were implemented with linear SVMs, a class of binary classifiers that finds the hyperplane that best separate training samples having different class memberships [31], the trade-off between model complexity and empirical error being determined by the constant complexity hyperparameter *C *> 0. However, regarding the construction of the ECOC coding matrix, we decided to use LDPC codes instead of random codes.

**Figure 2:**
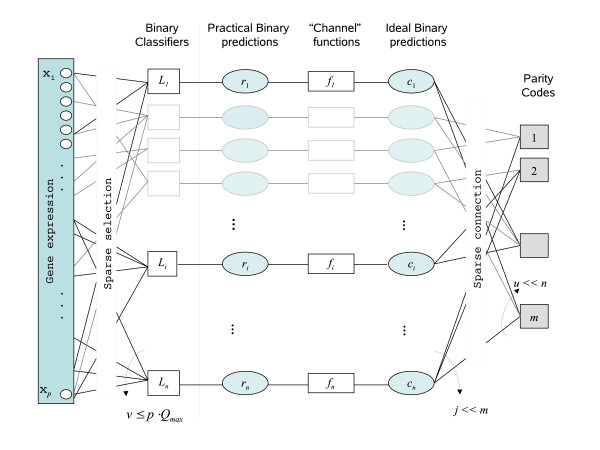
**The architecture of an ECOC-LDPC classifier under bounded gene selection**. Right squares represent constituent ECOC classifiers induced from simple parity codes, left squares represent practical binary classifiers, rectangles represent "channel" functions, ellipses represent binary predictions, and small circles represent gene expression measurements. Edges are put between constituent ECOC classifiers and ideal binary predictions *c_i _*taking care that the connectivity profile remains sparse (*j *< <*m*, *u *< <*n*). Ideal binary predictions *c_i _*and practical ones *r_i _*are constrained by "channel" functions *f_i _*modeling prior statistical knowledge about binary classifiers errors, *i *= 1,..., *n*. Each practical binary classifier *L_i _*selects a subset of *v *genes from a pool of *p *genes taking care that no more than $\frac{\left\lceil \log_{2}M\right\rceil }{n\cdot H\left(\frac{1}{p}\right)}$ genes get selected.

A key problem with conventional ECOC classifiers based on random codes is that randomness inhibits the systematic control of independence between binary classifiers as *n *approaches to infinity. A possible way to overcome this problem is to construct large ECOC classifiers from a number of small ECOC classifiers connected via shared binary classifiers. Small constituent ECOC classifiers able to locally control the key independence factor despite the size *n *of the overall ECOC classifier can be easily designed, for example with simple parity codes. Provided the connectivity profile of constituent ECOC classifiers and binary classifiers remains sparse, the overall ECOC design can be nicely interpreted in terms of the design of LDPC codes.

Briefly, LDPC codes are linear block codes obtained from sparse *random *bipartite graphs subject to sparsity constraints allowing a divide and conquer interpretation of generated ECOC classifiers[12]. Let *G *be a bipartite graph with *n *left nodes (called message nodes) and *m *right nodes (called check nodes). If the *n *message nodes are associated to the *n *coordinates of codewords **c **defined as those vectors (*c*_1_,..., *c_n_*) satisfying the constraint that the sum of the neighboring positions for all check nodes among the message nodes is zero, then *G *models a linear code of size *n *which can protect at least *k *= *n *- *m *bits of information and which structure can be dissected into *m *simple parity codes. In addition, if the connectivity profile of *G *is sparse, i.e., each codeword bit is constrained by *j *< <*m *parity codes and each parity code constraints *u *< <*n *codeword bits, then the corresponding linear code turns to be an LDPC code. The sparsity of the graph structure is a key property in the design of efficient LDPC decoding algorithms for a variety of channel models. A channel model subsumes our prior knowledge about the statistics of binary errors. In this paper, the iterative message passing decoding algorithm described in [13] for the Additive White Gaussian Noise channel is used. A factor graph [32] model of a typical LDPC code is shown in Figure 2. The construction of ECOC classifiers based on LDPC codes is straightforward once the bipartite graph model of the underlying LDPC code is given. In factor graph terms, we just need to associate right message nodes to ideal binary classifiers predictions *c_i _*and left check nodes to constituent ECOC classifiers constructed from simple parity codes. To complete the factor graph model of an ECOC-LDPC classifier, message nodes *r_i _*modeling practical binary classifiers predictions and check nodes *f_i _*modeling prior statistical knowledge about pairs (*c_i_*, *r_i_*) ("channel functions") must be introduced. A request for an ECOC prediction on a set of input features **x **starts with the computation of a corrupted codeword **r**(**x**) by the set of *n *binary classifiers. Assuming a suitable channel model specified by check nodes *f_i_*, the corrupted codeword **r**(**x**) is given to an iterative message passing decoding algorithm for the computation of a hopefully good estimate $\hat{c}(x)$ of the unknown codeword **c**(**x**) encoding the unknown class label *y *associated to **x**. Remarkably, the computation of $\hat{c}(x)$ can be fully described as a message passing algorithm over the ECOC-LDPC factor graph. In addition to convenient graphical $\hat{c}(x)$ computation, ECOC-LDPC factor graphs also allow for seamless integration of general bounded gene selection strategies. We just need to add message nodes *x_k_*, *k *= 1,..., *p*, modeling gene expression behavior, check nodes *L_i_*, *i *= 1,..., *n*, modeling practical binary classifiers and a sparse connectivity profile ensuring that at each *L_i _*the number *v *of incident edges (selected genes) is no more than $p\cdot Q_{max}\approx \frac{log_{2}M}{n\cdot H(\frac{1}{p})}$, in agreement with Eq.2.

### Microarray Datasets

Eight cancer microarray data sets were used in the evaluation of binary mediated multiclass classification with bounded optimum *S*2*N *gene selection. The **Lymphoma **dataset [33] consists of 62 samples of a specialized cDNA chip spanning *M *= 3 subtypes of Diffuse large B-cell lymphoma, each sample defined by the expression of *p *= 4026 genes. Samples in the Lymphoma dataset are highly imbalanced: 42 samples of diffuse large B-cell lymphoma, 9 of follicular lymphoma and 11 of chronic lymphocytic leukemia. Original data is available at http://llmpp.nih.gov/lymphoma/data/figure1. In this study, a preprocessed dataset version compiled by [34] based on [35] was used.

The Small Round Blue Cell Tumors (**SRBCT**) dataset [36] consists of 63 samples of a specialized cDNA chip spanning *M *= 4 subtypes of small round blue cell tumors of childhood, each sample defined by the expression of *p *= 2308 genes. Samples are distributed as follows: 12 samples of neuroblastoma, 20 samples of rhabdomyosarcoma, 8 samples of non-Hodgkin lymphoma and 23 samples of the Ewing family of tumors. In this study, a preprocessed dataset version available at http://research.nhgri.nih.gov/microarray/Supplement/index.html was used.

The **Brain **dataset [37] consists of 42 samples of the Affymetrix HuGeneFL chip spanning *M *= 5 tumors classes of the central nervous system, each sample defined by the expression of *p *= 5597 genes. Samples are distributed as follows: 10 medulloblastomas, 10 malignant gliomas, 10 atypical teratoid/rhabdoid tumors (AT/RTs), 8 primitive neuro-ectodermal, tumors (PNETs) and 4 human cerebella. In this study, the original dataset version (Dataset A) was used. Expression values based on average difference units were computed using the Affymetrix GENECHIP MAS 4.0 analysis software. This dataset is available at http://www.broadinstitute.org/mpr/CNS/.

The **NCI60 **dataset [35] consists of 61 samples of a specialized cDNA chip spanning *M *= 8 tumor classes, each sample defined by the expression of *p *= 5244 genes. Samples are distributed as follows: 7 breast, 5 central nervous system, 7 colon, 6 leukemia, 8 melanoma, 9 non-small cell lung carcinoma, 6 ovarian and 9 renal tumors. Original data is available at http://genome-www.stanford.edu/nci60. In this study, a preprocessed dataset version compiled by [34] based on [35] was used.

The **Staunton **dataset [38] consists of 60 samples of the Affymetrix Hu6800 chip spanning *M *= 9 classes of tumors, each sample defined by the expression of *p *= 5726 genes. Expression values based on average difference units were computed using the Affymetrix GENECHIP MAS 4.0 analysis software. In this study, a preprocessed dataset version compiled by [1] involving the rescaling of gene expression measurements to the interval 0[1] was used. This dataset is available at http://www.gems-system.org/.

The **Su **[39] consists of 174 samples of the Affymetrix U95a chip spanning *M *= 11 classes of tumors, each sample defined by the expression values of *p *= 12533 genes. Expression values based on average difference units were computed using the Affymetrix GENECHIP MAS 4.0 analysis software. In this study, a preprocessed dataset version compiled by [1] involving the rescaling of gene expression values to the interval 0[1] was used. This dataset is available at http://www.gems-system.org/.

The **GCM **dataset [18] consists of 190 samples of the Affymetrix Hu6800 and Hu35K chips spanning *M *= 14 tumor classes of primary tumors, each sample defined by the expression of values *p *= 16063 genes. Expression values based on average difference units were computed using Affymetrix GENECHIP MAS 4.0 analysis software. This dataset, which comes with a public train-test partition involving *q *= 144 samples for training and 46 for test, is available at http://www.broadinstitute.org/cgi-bin/cancer/datasets.cgi.

The **GCM RM **dataset [40] consists of 123 samples of the Affymetrix Hu6800 chip spanning *M *= 11 classes of tumors, each sample defined by the expression values of *p *= 7129 genes. This dataset was derived from the GCM dataset with the purpose of improving multiclass classification with variability estimates of repeated gene expression measurements. Hence, expression values were computed with the more robust log scale multi-array analysis (RMA) measure. This dataset, which comes with a public train-test partition involving *q *= 96 samples for training and 27 for test, is available at http://expression.washington.edu/publications/kayee/shrunken_centroid/.

### Experimental Protocol

Optimum bounded gene selection over OAA and ECOC multiclass based on linear SVMs classifiers was evaluated on 8 publicly available microarray datasets (M ∈ {3, 4, 5, 8, 9, 11, 14}). Aiming a systematic evaluation of the *n*-dimension, we restricted ourselves to the class of ECOC classifiers based on LDPC codes. For both OAA and ECOC classifiers, binary classifiers decisions were fusioned by means of soft-decoding techniques. Hence, OAA classifiers based on hinge loss decoding of SVM's outputs and ECOC classifiers based on LDPC codes able to perform soft iterative decoding of SVM's outputs were used. Owing to the constraint *p *> >*q*, which highly limits the diversity between induced binary classifiers, just one iterative decoding loop was allowed. The Java Weka library version 3.4.10 [41] was used to provide the implementations of OAA multiclass and binary linear SVM classifiers. An extension of the Weka library was developed to implement ECOC classifiers based on LDPC codes and bounded optimum gene selection for both OAA and ECOC classifiers.

#### Assessing the classification performance

The classification performance of OAA and ECOC multiclass classifiers was evaluated by means of a randomized strategy. Based on [42] and [35], 200 Montecarlo 4:1 ($\frac{4}{5}$ for training and $\frac{1}{5}$ for testing) partitions of available data were considered. For those datasets with a public train-test partition, the specific train-test evaluation was additionally performed. The following performance metrics were considered: the test error rate, the number of binary classifiers, the number of genes per binary classifier, the overall number of selected genes and the stability of gene selection. Briefiy, stability of gene selection measures how multiple classification models resemble between them; models may be close to each other in terms of error, but can be distant in terms of their forms (the identity of selected genes) [43]. Thus, stability of gene selection is an important requirement for ensuring reliable conclusions in microarray data analysis [44,45]. Stability of gene selection with respect to changes in the training data was measured by means of the Salton's cosine coefficient [46]. Let *A_i _*and *A_j _*respectively denote the sets of genes selected by classifier *A *in partitions *i *and *j*, *i *≠ μ*j*. Hence, the similarity between sets *A_i _*and *A_j _*according to the Salton's coefficient is given by $\frac{\#\text{​}genes\;in\;both\;A_{i}\;and\;A_{j}}{\sqrt{\#\text{​}genes\;in\;A_{i}}\cdot \sqrt{\#\text{​}genes\;in\;A_{j}}}$. Using 200 random train-test partitions lead to 200 · 199/2 pairwise similarity measurements from which the mean stability of gene selection can be reported.

#### Searching the best parameters

Regarding the honest computation [47] of best *n *and *Q*(*n*) parameters for ECOC classifiers, a two-stage optimization approach based on nested 10-Fold CV loops was performed. At each train-test partition, the constant complexity hyperparameter *C *of binary linear SVM classifiers was set to 1 and the best (*n*, *Q*(*n*)) pair was estimated by a nested 10-Fold CV error minimization loop in the current training dataset over the grid [*n_min_*, *n_max_*] × (0, *Q_max_*], *n_min _*= ⌈*log*_2_*M*⌉ + 2, *n_max _*= ⌈*η *·*log*_2_*M*⌉, *η *= 5, 10, 15. Regarding the exploration of the *Q *dimension, the *S*2*N *metric was used for inducing ordered lists of genes at each binary classifier. Briefiy, the class discrimination ability of the *j *- *th *gene at each binary classifier under the *S*2*N *metric, denoted as *S*2*N*(*j*), is defined as follows

(3) $$S2N(j)=\frac{\mu {\left(j\right)}_{+}-\mu (j)-}{\sigma {\left(j\right)}_{+}+\sigma (j)-}$$

where *μ*(*j*)_+_, *μ*(*j*)_- _and *σ*(*j*)_+_, *σ*(*j*)_- _denote the means and standard deviations of the *j *- *th *gene in positive and negative examples in the current (binary) training set. Most *g *important genes under the *S*2*N *metric are defined as the first *g*/2 and the last *g*/2 genes in the ranked list of genes. For a fixed number *n *of binary classifiers, optimum bounded gene selection requires the estimation of the optimum number of genes *g*(*n*), or its fractional equivalent $Q(n)=\frac{g(n)}{p}$, in the list of p* *Q_max_*(*n*) most important genes. Such threshold can be estimated by a nested 10-Fold CV loop in the current training set using the multiscale resolution approach described in the Results section. The process must be repeated for each candidate *n *in the range [*n_min_*, *n_max_*]. Afterwards, the best performing (*n*, *Q*(*n*)) pair can be reported. In case of multiple solutions, that involving the largest *n*, i.e., the smallest *Q*(*n*), is selected.

An additional nested loop of 10-Fold CV was performed to optimize the constant complexity hyperparameter *C *of linear SVMs. Although it would have been better to jointly optimize (*n*, *Q*(*n*), *C*), this would have been computationally prohibitively expensive. Alternatively, the two-step optimization strategy described in [9] was used. Hence, we first set (*n*, *Q*(*n*)) at the best pair of values found at *C *= 1, and then decreased and increased *C *until no improvement was observed for three consecutive steps in nested 10-Fold CV loops. The best performing *C *along with the best performing (*n*, *Q*(*n*)) pair at *C *= 1 were then used as input parameters for the construction of the best ECOC classifier on the current training set and its posterior evaluation on the testing set. Notice that the final performance estimate obtained by this procedure is selection-bias free because each original testing set is used only once to estimate the performance of a single classification model that was built by using training data exclusively. Except for the preselection of *n *= *M*, a similar approach was used to estimate the best *Q*(*M*) and the best *C *for OAA classifiers. Table 5 shows the central tendency and the variation of the best *C *for ECOC and OAA classifiers over 200 Montecarlo 4:1 train-test partitions. Results suggest that *C *= 1 is indeed a reasonable initial guess.

**Table 5:** The best *C *for ECOC and classifiers based on linear SVMs

Dataset	ECOC at *η *= 5^a^	ECOC at *η *= 10^a^	ECOC at *η *= 15^a^	OAA^a^
Lymphoma	NA	1:1-1	1:1-1	1:1-1
SRCBT	1:1-1	1:1-1	1:1-1	1:1-1
Brain	1:1-1	1:1-1	1:1-1	1:1-1
NCI60	1:1-1	1:1-1	1:1-1	1:0.5-1
Staunton	1:1-1	1:1-1	1:1-1	1:0.5-1
GCMRM	1:1-1	1:1-1	1:1-1	1:1-1
Su	1:1-1	1:1-1	1:1-1	1:0.5-1
GCM	1:1-1	1:1-1	1:1-1	1:0.5-1

The best *C *for ECOC classifiers of size at most ⌈*η*·*log*_2_*M*⌉ and OAA classifiers, both based on linear SVM classifiers, under bounded optimum *S*2*N *gene selection over 200 Montecarlo 4:1 train-test partitions

^a ^The best *C *expressed as median: lower quartile-upper quartile.

#### Assessing the statistical significance of results

To assess the statistical significance of observed differences between performance measures of ECOC and OAA classifiers, we invoke the concept of first order stochastic dominance [48] developed in the context of international economics [49]. Let *F *and *G *denote the cumulative distribution functions of two comparison groups regarding the study of some performance measure, e.g., the gene selection stability of ECOC and OAA classifiers. First-order stochastic dominance of *F *with respect to *G *is defined as *F *(*z*) - *G *(*z*) ≤ 0 uniformly in *z *∈ ℜ, with strict equality for some *z*. Since this considers all moments of the distributions, it is a stricter test of stability differences than just comparing mean levels of stability. In order to implement first-order stochastic dominance analysis, nonparametric two-sided and one-sided Kolmogorov-Smirnov (KS) tests [50] will be used. The KS test looks for differences in two distributions, both in terms of shape and location. Although the KS test has good power for testing general differences in distributions and not just in their central tendencies, it is less sensitive than the t-test if data is normal. Considering this issue, normality of distributions was analyzed first by means of the Shapiro-Wilk test [50,51]. The two-sided KS statistic tests the hypothesis that both distributions are identical; the null and alternative hypotheses can be expressed as:

(4) $$H_{0}:F(z)-G(z)=0\forall z\in ℜ\;\text{vs}\;H_{1}:F(z)-G(z)\neq 0\;\text{for}\;\text{some}\;z\in ℜ$$

By contrast, the one-sided test of stochastic dominance of *F *over *G *(the distribution associated with *F *lies to the right of that associated with G) can be formulated as:

(5) $$H_{0}:G(z)-F(z)\geq 0\;\forall z\in ℜ\;\text{vs}\;H_{1}:G(z)-F(z)<0\;\text{for}\;\text{some}\;z\in ℜ$$

Similarly, the one-sided test of stochastic dominance of *G *over *F *(the distribution associated with *F *lies to the left of that associated with G) can be formulated as:

(6) $$H_{0}:F(z)-G(z)\geq 0\;\forall z\in ℜ\;\text{vs}\;H_{1}:F(z)-G(z)<0\;\text{for}\;\text{some}\;z\in ℜ$$

Hence, in order to conclude that *F *(*G*) stochastically dominates *G *(*F *) we need to reject the null hypothesis for the two sided test, but not reject the null for the corresponding one sided test. The test statistics for the two and one sided tests are, respectively:

(7) $$D=\underset{1\leq i\leq N}{\max}|F_{u}(z_{i})-G_{v}(z_{i})|$$

(8) $$D^{+}=\underset{1\leq i\leq N}{\max}\;\{G_{v}(z_{i})-F_{u}(z_{i})\}$$

(9) $$D^{-}=\underset{1\leq i\leq N}{\max}\;\{F_{u}(z_{i})-G_{v}(z_{i})\}$$

where *u *and *v *respectively denote the sample sizes from the empirical distributions of *F *and *G *and *N *= *u *+ *v*.

Hence, to test whether ECOC classifiers can attain better classification performance than OAA classifiers, the two-sided *D *(Eq. 7) and the one-sided *D^- ^*(Eq. 8) statistics were used (the *alternative *parameter of the *ks.test *function in the *stats *R package respectively set to "two.sided" and "less"). A similar approach was used to assess the statistical significance of the differences between the overall fraction of selected genes by ECOC and OAA classifiers. Finally, to assess the statistical significance of stability differences between ECOC and OAA classifiers, the *D *(Eq. 7) and the *D*^+ ^(Eq. 9) statistics were used (the *alternativ*e parameter of the *ks.test *function in the stats R package respectively set to "two.sided" and "greater"). One-sided KS tests were supplemented with one-sided Mann-Whitney U tests (MW) for analyzing the difference between medians of two groups. A criterion alpha level of 0.05 was used for all statistical tests.

## Appendix

### A more formal derivation of an upper bound for the number of genes per binary classifier

We consider the problem of designing accurate and sparse binary mediated multiclass classifiers for microarray data samples. In this context, accuracy is mainly determined by the power of the error correction code defining the multiclass to binary mapping and sparsity is mainly determined by the efficacy of gene selection algorithms used at the binary classification level. A natural question that arises in this system is what amount of information genes can transfer to the multiclass classifier output as the number *p *of genes grows. Knowing such limitation may play a crucial role in the design of effective gene selection algorithms which could significantly reduce their search spaces. Shannon's Information Theory concepts [52] can provide some useful insights into this fundamental question. In particular, the concept of mutual information (MI) can be used to evaluate the information content of a subset of genes with regard to individual binary output classes and the information content of a set of binary output classes with regard to the target multiclass output class. The use of MI for general multiclass classification problems can be motivated by Fano's inequality [53] which gives a lower bound for the probability of error *p_e _*when estimating a discrete random variable *y *∈ {*c*_1_,..., *c_M_*} from another random variable x ∈ ℜ*^p ^*as a function of their MI *I*(*y*, x)

(10) $$p_{e}\geq \frac{H(y)-I(y;\text{x})-1}{log_{2}M}$$

Where $p_{e}=P(\hat{y}\neq y)$, *H*(*y*) is the Shannon entropy of *y*, $\hat{y}=g(x)$ is a discrete random variable used to estimate *y *and $y\to x\to \hat{y}$ is the Markov Chain modeling the overall classification process. Let us now consider Markov Chains $\text{y}\to \text{x}\overset{T_{i}}{\to }{\text{v}}_{i}\overset{L_{i}}{\to }r_{i}$ and *y *→ **x **→ **r **modeling the prediction of a target output class *y *∈ {*c*_1_,..., *c_M_*} from genes **x **∈ {0, 1}*^p ^*by the mediation of binary output classes **r **= (*r_i_*), each *r_i _*modeling the binary output class of a classifier *L_i _*on subset of genes **v***_i _*∈ {0, 1}*^g^*, *g *<*p*, extracted by a gene selection algorithm *T_i _*on genes **x**, *i *= 1,..., *n*. By the Fano's inequality, minimizing *p_e _*requires the maximization of *I *(*y*, **x**) = *H *(*y*) - *H *(*y *| **x**). Since *y *is fixed, we have *I*(*y*, **x**) ≤ *H*(*y*) ≤ *log*_2_*M*. On the other hand, by the data processing inequality [54], we have *I *(*y*, **r**) ≤ *I *(*y*, **x**). In other words, the maximization of *I *(*y*, **x**) requires the choice of an error correcting output code such that *I *(*y*, **r**) is maximized. In addition, let **r **be a set *n *i.i.d. random variables *r_i_*. Thus, we have *I*(*y*, **r**) = Σ*_i _I*(*y*, *r_i_*) and $I(y,r_{i})\leq \frac{log_{2}M}{n}$. Again by the data processing inequality, we have *I*(**v***_i_*, *r_i_*) ≤ *I*(*y*, *r_i_*). If we further assume that *T_i _*is a gene selection algorithm able to select just relevant genes to *r_i_*, i.e., *H*(**v***_i _*| *r_i_*) = 0, we have *I*(**v***_i_*, *r_i_*) = *H*(**v***_i_*) - *H*(**v***_i _*| *r_i_*) = *H*(**v***_i_*). Finally, let genes in **v***_i _*be a set of *g *i.i.d. binary random variables. Thus, we have *H*(**v***_i_*) = *H*(*T_i_*(**x**)) = *Q *· *p *· *H*(*f*) where *Q *is the fraction of relevant genes to *r_i _*and *H*(*f*) is the binary entropy function measuring the information content of a generic gene which is expressed with probability *f *and not expressed with probability 1 - *f*. Hence, the following upper bound on the fraction of genes *Q *that can be handled by any binary classifier in a binary mediated multiclass classifier for microarray data samples is obtained

(11) $$Q\leq \frac{log_{2}M}{p\cdot n\cdot H(f)}$$

## Authors' contributions

ET devised the study, set up and performed simulation experiments, and drafted the manuscript. LO contributed to the design of simulation experiments, to the statistical analysis of experimental results and to the manuscript. PB and LA contributed to the design of simulation experiments, to organize experimental results and to the manuscript. All authors read and approved the final manuscript.
